# Changing Trends of Breast Cancer Survival in Sultanate of Oman

**DOI:** 10.1155/2011/316243

**Published:** 2010-10-25

**Authors:** Shiyam Kumar, Ikram A. Burney, Adel Al-Ajmi, Mansour S. Al-Moundhri

**Affiliations:** ^1^Medical Oncology Unit, Department of Medicine, College of Medicine and Health Sciences, Sultan Qaboos University (SQU), P.O. Box 35, Muscat 123, Oman; ^2^Department of Surgery, College of Medicine and Health Sciences, Sultan Qaboos University (SQU), Muscat, Oman

## Abstract

Breast cancer is the leading cause of cancer-associated mortality in women, with elevated incidence in developing countries. This retrospective study included all 122 patients diagnosed with breast cancer from January 2003 to December 2008 in the Sultanate of Oman. Age at presentation was 47.41 years (SD±12.88), with one-third of patients younger than 40 years. The majority of patients presented with stage III (41.2%) and IV (18.2%) breast cancer. T size (*P* = .023), skin involvement (*P* = .003), and stage at presentation (*P* = .004) were significantly associated with overall survival. Skin involvement at presentation (*P* = .003), T size (*P* = .09), lymph node status (*P* = .013), and stage (*P* = .003) were strong predictors of relapse-free survival. Patients had a 5-year survival of 78%, compared to 64% of breast cancer patients diagnosed between 1996 and 2002 identified in our previously published study. Thus, despite Omani breast cancer patients continuing to present with advanced breast cancer, survival rates have significantly improved.

## 1. Introduction

Breast cancer is the leading cause of cancer-related mortality in women worldwide. Almost half of annually diagnosed females with breast cancer belong to developing countries, where they present at a younger age with advanced-stage disease. These women also have poor overall outcomes compared to women in developed countries. The advanced stage of presentation of breast cancer in developing countries was attributed to a lack of mass education and screening programs, poverty, poor access to health care facilities, lack of expertise, and poor country infrastructure [1–7].

It is an established fact that ethnic disparities affect breast cancer outcome. Despite correction of well-known factors associated with breast cancer-related outcome, such as tumor size, lymph node status, hormone receptor expression, Her2/neu gene expression, stage, and age at presentation, racial differences were prominent as prognostic factors and have been associated with genetic differences between races. Investigators have proposed multiple reasons to explain these differences between races [1, 8–12]. 

The Sultanate of Oman is a developing Asian country in the Gulf Region with a developing health care system. Like women worldwide, Omani women also share major burden of breast cancer incidence and associated mortality. One out of five Omani women is diagnosed with breast cancer in her lifetime, and the age-standardized incidence rate is 15.6 per 100,000. In our last reported study, we found that age at diagnosis is younger in Oman than in the western world, and the majority of patients present at advanced stages of disease (III and IV) [1]. 

In our last study, we reported the clinicopathologic features, such as treatment modalities, outcome, and associated prognostic factors for Omani women, that have a diagnosis of breast cancer between the years of 1996 and 2002. The results of this previous study revealed that patients in Oman presented at a younger age and with an advanced stage of disease. Furthermore, there is an underutilization of neoadjuvant (NA) therapy with 5-year relapse-free survival (RFS) and 5-year overall survival (OS) of 64% and 62%, respectively [1]. In this present paper, we analyzed data retrospectively to determine if OS had improved. We also analyzed whether the trends of disease presentation or associated outcome had changed between 1996–2002 and 2003–2008.

## 2. Patients and Methods

We analyzed patient data using the computerized hospital information system of our university hospital for patients admitted with the diagnosis of invasive breast cancer from January 2003 to December 2008. Our hospital (Sultan Qaboos University Hospital) is one of the two main hospitals providing cancer treatment in the Sultanate of Oman. Among the patients included in this retrospective data, the majority were diagnosed and treated in this hospital, but some patients presented either after being diagnosed in other hospitals or after undergoing surgery at peripheral hospitals. Our pathology department reviewed almost all histopathological specimens for confirmation of diagnosis and immune staining of tissue for estrogen receptor (ER), progesterone (PgR), and Her2/neu status. Due to the advanced stage of breast cancer at presentation, the breast cancer stage in most patients was determined by CT scans of the chest, abdomen, and pelvis, as well as with bonescans.

The records of all patients with a confirmed diagnosis of invasive breast cancer were reviewed, and a database was created. Variables were identical to those presented in our previous published study and included age and sex; date of diagnosis; side of involved breast; histopathological type of tumor; clinical and pathological tumor size; pathological or clinical involvement of skin or nipple areola complex; clinical and pathological lymph node involvement; tumor grade; marker status of tumor, including ER, PgR, and Her2/neu status; clinical and pathological stage of the patients. Records were also reviewed for the date of last followup exam, date and site of relapse, and date of death, when relevant. 

Relapse-free survival (RFS) was measured from diagnosis to the date of documented relapse and was censored at the date of last followup. Overall survival (OS) was measured from the date of diagnosis to the date of death and censored by the last date of followup. 

Kaplan-Meir curves were used to determine OS and RFS and the log-rank test was used for comparison analysis. The Cox proportional hazard model was used for univariate analysis with the variables included being age, menopausal status, tumor size, lymph node status, tumor grade, and estrogen receptor status. The Cox model was used for multivariate analysis including all statistically significant factors as per univariate analysis. All reported *P*-values herein are nominal 2-sided. Data analysis was performed using SPSS version 16.

## 3. Results

### 3.1. Clinical and Pathological Features

A total of 122 patients were identified with a diagnosis of invasive breast cancer. The majority of patients were of Omani origin 108 (88.5%). The mean age of all patients was 47.41 (SD ± 12.88) years and 3 of 122 patients were male (Table 1). Almost one-third (32%) of the patients were younger than 40 years of age at the time of diagnosis and 55.7% were premenopausal. More than half (55.7%) of patients underwent nonbreast conserving surgery. Twenty-nine patients (23.7%) received neoadjuvant (NA) chemotherapy, which is equivalent to 47% of the patients who presented with locally advanced disease stage (stage IIB to IIIC). Of the total, 38% of tumors were negative for hormone receptor (ER and PgR) expression, and 21% were positive for the Her2/neu gene detected by immunohistochemistry. An additional 15% of patient data regarding Her2 status were missing. 

External beam radiotherapy was administered to 73.8% of patients. All patients were treated locally, differing from our previous paper due to the fact that radiation facilities were not available in Oman at the time. 

Table 2 summarizes the patients' clinical and pathological stage of breast cancer. Mean clinical tumor size was 5.3 cm (±2.7 cm), while mean pathological size was 3.8 cm (±2.7 cm) which is almost identical to our previous paper (5.4 cm (S.D. 3.86) and 4.6 cm (S.D. 3.29), resp.). Forty-four patients (36%) had a tumor size of >5.5 cm. Among those 44 patients with large tumor size, 34 patients presented with clinical T4 disease, versus only 9 patients who presented with a clinical T1 lesion (38 and 22 patients, resp., were reported to have clinical T4 and T1 lesions, resp., in our last paper). More than half of patients presented with advanced disease, with stages III and IV diagnosed in 41.2% and 18.5% of patients, respectively (34.9% and 15.8%, resp., during years 1996–2002). Of the 89 patients (73% of total) who underwent axillary lymph node dissection, including 29 patients after NA chemotherapy, 18 (20%) had N3 (≥10 positive lymph nodes) disease, while 18 (20%) and 19 (21%) patients had N2 (4 to 6 positive lymph nodes) and N1 (1–3 positive lymph nodes), respectively. Among the 29 patients who received NA chemotherapy, 9 (31.0%) patients showed complete pathological response in the primary lesion and axillary lymph nodes (pCR), and 18 patients had N0 upon pathological exploration. All patients who were treated with neoadjuvant chemotherapy received anthracyclines followed by taxanes and trastuzumab where indicated, which resulted in significant pathological responses and reason for better outcome than our previous study as patients were treated with anthracycline or CMF- (cyclophosphamide, methotrexate, and fluorouracil) based regimens in the past paper. Ductal carcinoma was a major histopathological subtype, identified in 120 (98.4%) patients, with lobular carcinoma and carcinosarcoma identified in one patient each. Grade III disease was identified in 43 (35.2%) patients, while 60 (49.2%) and 10 (8.2%) patients had grade II and grade I differentiation, respectively (in 35.5%, 48.1%, and 16.4% patients, respectively, during years 1996–2002 as reported in our last paper). Information was missing for the remaining nine patients. Hormone receptor status was available for 118 (96.7%) patients and, among those, 71 (60.2%) and 74 (62.7%) patients expressed estrogen and progesterone receptors, respectively. Information regarding Her2/neu status was available for 103 (84.4%) patients, revealing that 26 (21.3%) had Her2 positive disease. Of all the 26 patients who were positive for Her2/neu gene, 21 received trastuzumab. Seven patients were treated in neoadjuvant setting with pCR in 2 patients and more than good partial response in the remaining five. Five patients received trastuzumab in palliative setting for stage IV disease while the remaining 9 patients were treated with trastuzumab in adjuvant setting.

### 3.2. Survival and Prognostic Factors

With a mean followup duration of 54 months, 27 patients died, and 4 patients were lost to followup. Among the patients that died, 10 deaths were of the metastatic group and 17 were of the nonmetastatic group. Among the 33 patients who experienced a relapse, 18 of those patients subsequently died from their disease. In nine patients, disease relapse led to bone metastases, and five patients had brain metastases; lungs, pleura, liver, and local relapse were also manifestations identified at the time of disease recurrence. 

Seventy-six patients were living at the end of the study, with no evidence of disease. Additional 15 patients have experienced persistent disease, with 12 of those 15 patients belonging to the metastatic group.

Skin involvement at presentation (*P* = .003), T size (*P* = .023), and stage (*P* = .004) were significant factors associated with OS as determined by univariate analysis. Additionally, skin involvement at presentation (*P* = .003), T size (*P* = .09), lymph node status (*P* = .013), and stage (*P* = .003) were strong predictors associated with RFS (Table 3). Stage at presentation was the only significant factor (*P* = .006) for OS, as determined by multivariate Cox regression analysis. 

**Figure 1:**
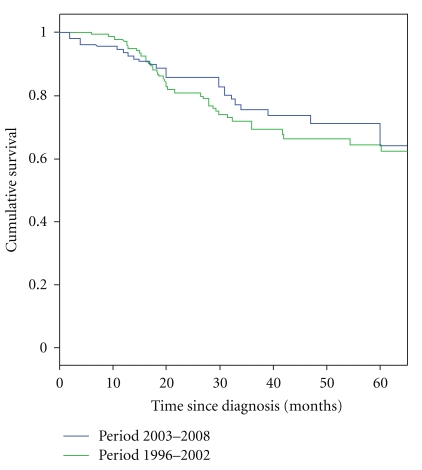
5-year overall survival curves in patients diagnosed with breast cancer in two time periods 2003–2008 or 1996–2002.

**Figure 2:**
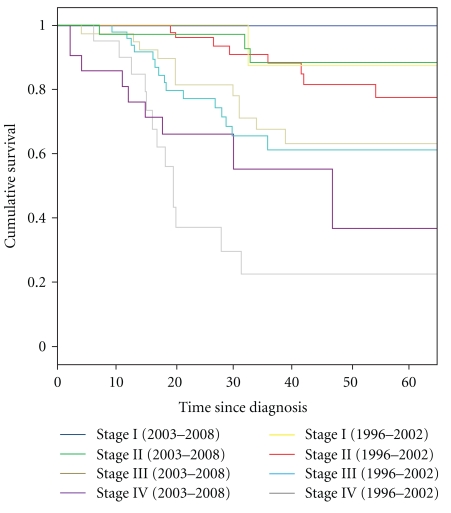
5-year overall survival curves per stage at diagnosis for those diagnosed in between (2003–2008) or (1996–2002).

The 5-year OS for all patients was 78% (Figure 1). The OS rates per stage were 100%, 87%, 62%, and 38% for patients for stages I, II, III, and IV, respectively (Figure 2), which is better in comparison with our previous paper in which 5-year cumulative survival for patients presenting with stage I, II, and III was 88%, 75%, and 59%, respectively (Figures 1 and 2).

## 4. Discussion

The risk factors associated with poor outcome of breast cancer such as young age at presentation, advanced stage, and negative hormone receptor status have been well recognized. In addition to these well-established risk factors, quality of provided care, health awareness, access to the health care system, and sociocultural beliefs are also closely linked to the ultimate outcome of disease [1–3, 6, 7, 10, 12]. This paper enables the comparison with the conclusions formulated in the previous paper. We can thus analyze the changes in breast cancer patients with regards to presentation of clinical and pathological features, treatment modalities used, and outcome.

In total, 122 patients were diagnosed and treated for invasive breast cancer in Oman. Consistent with the previous published paper, we observed that the age at presentation was still quite young, with a mean age of 47.41 (±12.88) years. Significantly, this age at presentation is almost a decade younger than women who present with invasive breast cancer and are from developed countries. In contrast, the age at presentation in the present study is relatively consistent between Oman and other developing countries, including neighboring Arab countries [2, 4, 6, 7, 13–15]. However, It should be highlighted that the presentation of breast cancer at younger age in the developing world may be due to younger population age distribution compared to Western countries. The majority of patients in this study were premenopausal, with 32% of women younger than 40 years of age and most having an advanced stage of disease at the time of diagnosis. Furthermore, only 8% of patients presented with a tumor smaller than 2 cm, which is also in contrast to the data from affluent countries, but consistent with data from neighboring regional and other developing countries [2–4, 6, 7, 10, 13–15]. The mean age at presentation in this present paper is a year younger compared to our previously published paper. Furthermore, fewer patients presented with stage I disease in this present paper, versus the previous paper (8% versus 14.5%) [1]. More than one-third (35.2%) of patients had tumors that were highgrade and negative expression of hormone receptors (40%), both of which are factors contributing to the aggressive nature and poor disease-associated outcome [2].

NA chemotherapy administered to patients for locally advanced breast cancer is generally accepted as the treatment of choice. This type of treatment is the reason for the increasing number of breast-conserving surgeries (BCSs) and is also associated with better OS [16–20]. However, data from various papers regarding the treatment of breast cancer in developing countries clearly demonstrates the underutilization of BCS. Furthermore, most patients undergo unwarranted surgeries early in the course of the disease at peripheral hospitals or have an advanced stage of tumor growth [1, 2, 6, 7, 13, 14, 16]. Similar to our previous published paper, the use of NA therapy was underutilized. However, the use of NA therapy did show some improvement, as in the present study, 29 patients (23.7%) were treated with NA chemotherapy, which accounts for 23.7% of total patients and 47% of patients who presented with locally advanced disease stage (stage IIB to IIIC). All those patients who received NA chemotherapy showed an excellent response, with 31% pCR in primary breast lesion and 62% pCR in recovered nodes. Adjuvant chemotherapy was administered to 95% of patients, a rate which is significantly improved over the previous study, in which only 60.2% of patients received adjuvant therapy.

We noticed better 5-year survival in patients of this present study, in which patients had a 5-year OS of 78% as compared to 64% in our last study [1] though disease stage at presentation was almost identical and reasons for better outcome are most likely due to introduction of frequent use of taxanes and trastuzumab along with aromatase inhibitors which were used very infrequently for patients reported previously (Figure 2). The 5-year OS is similar or better in comparison to studies regarding the efficacy of breast cancer treatment reported from other regional or developing countries [2, 6, 13]. Racial differences are now a known factor for breast cancer-related clinical outcomes, excluding other risk factors, as reported by two large database American studies. O'Malley and colleagues studied racial disparities affecting breast cancer-related clinical outcomes among white Asian, Hispanic, and African females diagnosed in California. The results of this study revealed significant differences in the 5-year survival rate between these groups [12]. Furthermore, Chu and coworkers reported the same survival differences in young black and white American females [9]. In both of these studies, besides differences attributed to race, investigators uncovered significant associations between socioeconomic and education status of the patients as well. In addition to other established risk factors of poor clinical outcome, stage at presentation has a very significant impact on OS. Patients who present with stage IV breast cancer have an almost 14-fold increase in risk of death, compared to patients diagnosed with stage I disease [12]. This data is consistent with our study, which reveals that patients who presented with stage I disease had a 5-year OS of almost 100%, versus 38% for patients with stage IV disease at presentation. Significantly, the 5-year OS for stage IV disease is almost twice as high as reported in our previously published study regarding the OS for metastatic disease [1].

Presentation at an advanced stage is common among patients with breast cancer in undeveloped countries. Socioeconomic issues, cultural barriers, and low literacy rates have been reported as the factors responsible for advanced stage of presentation, in addition to lack of screening programs and poor access to health care facilities [1, 5, 6, 11, 14, 15]. 

 In conclusion, although the number of patients in this study is relatively small, the study results show that patients in Oman still present with advanced stages of disease at a relatively young age. However, breast cancer patients enrolled in the present study have markedly improved RFS and OS. The improvement in RFS and OS is most likely due to utilization of various treatment modalities, including updated chemotherapy protocols and the use of trastuzumab. Further comparisons between this and the previous study reveal that Omani breast cancer patients still present with advanced disease, poor tumor differentiation, at a young age, and have a low percentage of hormone-positive tumors, all of which are known factors associated with poor overall disease outcome. Mass education programs, health awareness measures, and establishing screening programs are basic ways to decrease the disease burden and enable diagnosis at earlier stages of disease.

## Figures and Tables

**Table 1:** Clinical features and treatment modalities used for all 122 patients with invasive breast cancer in Oman between January 2003 and December 2008.

Clinical characteristics	Number	Percentage	Number	Percentage
	Period 2003–2008		Period 1996–2002	
Gender				
Female	119	97.5	**150**	**98.7**
Male	3	2.5	**2**	**1.3**
Age				
≤40	39	32	**31**	**20.4**
41–50	36	29.5	**46**	**30.3**
51–60	31	25.4	**49**	**32.2**
>60	16	13.1	**26**	**17.1**
Menopausal status (women)				
Premenopausal	68	57.1	**72**	**48.0**
Menopausal	51	42.8	**78**	**52.0**
Side of involved breast				
Left	65	53.2	**74**	**48.7**
Right	54	44.3	**76**	**50.0**
Bilateral	3	2.5	**2**	**1.3**
Surgery				
Modified radical mastectomy	68	55.7	**100**	**65.8**
Breast conservation surgery	43	35.2	**40**	**26.3**
Lumpectomy or biopsy only	—	—	**12**	**7.9**
Surgery not done (patient refusal or stage IV disease)	11	9	—	—
Chemotherapy*				
Neoadjuvant^*¶*^	29	23.7	**20** ^¤^	**13.2**
Adjuvant	65	53.2	**65 (17)**	**42.8**
FEC	26	40	**44 **(13)^≠^	
AC → Docetaxel ± trastuzumab	24	37	**4**	
Miscellaneous (AC, Paclitaxel, TAC, or CMF)	15	23	**11 (4)**	
Palliative	18	14.75	—	—
Chemotherapy refused	7	5.73	—	—
Missing information	3	2.45	—	—
Radiotherapy	90	73.8	**96**	**63.1**
Hormone treatment				
Tamoxifen or Aromatase inhibitors	85	69.7	**115 **(14)^§^	**75.7 (9.3)**

*A = Adriamycin, C = Cyclophosphamide, E = Epirubicin, F = Fluorouracil, M = Methotrexate, T = Docetaxel. ^*¶*^AC/FEC followed by paclitaxel or docetaxel  ±  trastuzumab, where indicated.

^¤^All received anthracycline (AC/FEC/FAC) regimens, ^≠^The numbers in brackets refer to patients with metastatic disease treated with chemotherapy and/or hormonal treatment. All these patients were treated with anthracycline-based regimens (AC/FEC/FAC). All but 3 patients were treated with tamoxifen; aromatase inhibitors were not in use during that period. 3 patients were treated with goserelin.

**Table 2:** Clinical and pathological staging of breast cancer.

	Clinical stage*		Clinical stage (*N* = 152)		Pathological stage*		Pathological stage^†^ (*N* = 120)	
	Number	Percentage	Number	Percentage	Number	Percentage	Number	Percentage
	Period 2003–2008		Period 1996–2002		Period 2003–2008		Period 1996–2002	
Primary tumor								
Tis	—	—	**2**	**1.3**	—	—	**2**	**1.7**
T0	—	—	—	—	9	7.4	—	—
T1	9	8.4	**22**	**14.5**	19	18.4	**12**	**10.0**
T2	43	40.1	**64**	**42.1**	48	46.6	**66**	**55.0**
T3	21	19.6	**38**	**25.0**	27	26.2	**32**	**26.7**
T4	34	31.7	**26**	**17.1**	—	—	**8**	**6.6 **

Node								
N0 (*p* ^*¶*^ = 0)	26	24.5	**94**	**61.8**	44	41.5	**37**	**30.8**
N1 (*p* = 1–3)	33	31.1	**36**	**23.7**	24	22.6	**40**	**33.3**
N2 (*p* = 4–9)	29	27.3	**12**	**7.9**	18	17.0	**29**	**24.2**
N3 (*p* ≥ 10)	18	17	**10**	**6.6**	20	18.9	**14**	**11.7 **

Stage								
0	—	—	**2**	**1.3**	8	8.0	**2**	**1.7**
I	7	6	**13**	**8.6**	10	10.0	**12**	**10.0**
II	46	39	**60**	**39.5**	39	38.0	**56**	**46.7**
III	43	36	**53**	**34.9**	41	41.0	**50**	**41.7**
IV^*∞*^	22	18	**24**	**15.8**	—	—	—	—

*Data is not available for all patients in all categories, as some patients presented to this hospital after surgical intervention at peripheral hospitals, and some of patients with stage IV disease did not undergo surgical intervention. ^*¶*^Denotes pathological nodal staging. ^*∞*^Only clinical stage is shown for stage IV patients. ^†^Pathological staging did not include neoadjuvant chemotherapy patients (*N* = 20) or those with metastatic disease who did not have breast surgery (*N* = 12).

**Table 3:** Overall survival in all patients (*N* = 122) and relapse-free survival for patients in the nonmetastatic group (*N* = 100). Univariate analysis performed using Cox proportional hazard's model.

Variable	Overall Survival		Relapse-Free Survival	
	Risk ratio	*P*-value	Risk ratio	*P*-value
Skin involvement at presentation				
Negative	1		1	
Positive	3.3 (1.5–7.5)	.003	3.0 (1.4–6.4)	.003

Pathological T-size				
T1 + T2	1		1	
T3 + T4	2.6 (1.1–6.1)	.023	1.9 (0.9–3.9)	.09

Stage				
I and II	1		1	
III /IV	5.4 (1.8–15.8)	.004	3.7 (1.6–8.6)	.003

Lymph nodes involved				
Negative	—	—	1	
Positive	—	—	2.9 (1.3–7.1)	.013
